# The Effects of Geometric Features of Intraluminal Thrombus on the Vessel Wall Oxygen Deprivation

**DOI:** 10.3389/fbioe.2022.814995

**Published:** 2022-03-28

**Authors:** Burton Carbino, Alexander Guy, Michael Durka, Rana Zakerzadeh

**Affiliations:** ^1^ Department of Engineering, Rangos School of Health Sciences, Duquesne University, Pittsburgh, PA, United States; ^2^ NASA Jet Propulsion Laboratory, California Institute of Technology, Pasadena, CA, United States; ^3^ Department of Mechanical Engineering and Materials Science, Swanson School of Engineering, University of Pittsburgh, Pittsburgh, PA, United States

**Keywords:** abdominal aortic aneurysm, intraluminal thrombus, geometric modeling, parametric study, oxygen transport, hypoxia

## Abstract

The objective of this paper is to analyze the association of intraluminal thrombus (ILT) presence and morphology with oxygen transport in abdominal aortic aneurysms (AAA) and local hypoxia. The biomechanical role of the ILT layer in the evolution of the aneurysm is still not fully understood. ILT has been shown to create an inflammatory environment by reducing oxygen flux to the arterial wall and therefore decreasing its strength. It has been also hypothesized that the geometry of the ILT may further affect AAA rupture. However, no previous research has attempted to explore the effect of morphological features of ILT on oxygen distributions within the AAA, in a systematic manner. In this study, we perform a comprehensive analysis to investigate how physiologically meaningful variations in ILT geometric characteristics affect oxygen transport within an AAA. We simulate twenty-seven AAA models with variable ILT dimensions and investigate the extent to which ILT attenuates oxygen concentration in the arterial wall. Geometric variations studied include ILT thickness and ILT length, as well as the bulge diameter of the aneurysm which is related to ILT curvature. Computer simulations of coupled fluid flow-mass transport between arterial wall, ILT, and blood are solved and spatial variations of oxygen concentrations within the ILT and wall are obtained. The comparison of the results for all twenty-seven simulations supports the hypothesis that the presence of ILT in AAA correlates to significantly impaired oxygen transport to the aneurysmal wall. Mainly, we observed that ILT thickness and length are the parameters that influence decreased oxygen flow and concentration values the most, and thick thrombi exacerbate hypoxic conditions in the arterial wall, which may contribute to increased tissue degradation. Conversely, we observed that the arterial wall oxygen concentration is nearly independent of the AAA bulge diameter. This confirms that consideration of ILT size and anatomy is crucial in the analysis of AAA development.

## Introduction

Abdominal Aortic Aneurysm (AAA) is characterized by the continuous and gradual dilation of the infrarenal aorta which results from the degradation of the extracellular matrix in the arterial wall (Jana et al., 2019). The prevalence of AAA in general population ranges from 1.0 to 14.2% in men and from 0.2 to 6.4% in women (Li et al., 2013). The majority of AAAs are asymptomatic and acute AAA ruptures are estimated to cause 4–5% of sudden death in developed countries, and is the 14th leading cause of death in the United States (Aggarwal et al., 2011). Upon rupture, this disease has a high mortality rate of 50–80%, and about 50% of patients die before ever reaching the hospital due to the asymptomatic nature of AAA. However, the processes associated with AAA development and evolution are still not fully understood. It is imperative to thoroughly understand the conditions under which AAA rupture to enhance development of more effective treatments leading to improved patient outcome. Although some clinical evidence suggests that aneurysm diameter is positively associated with the risk of rupture, it has been observed that this maximum diameter criterion may not be appropriate, since sometimes smaller AAA rupture while larger diameter aneurysms remain intact and asymptomatic (Vorp and Geest, 2005). Therefore, other parameters may also play a role in causing or predisposing the AAA to rupture. One possible reason for this variability in clinical outcomes is the formation of a layer called intraluminal thrombus (ILT). Diverse findings from the literature regarding the role of ILT in AAA progression are discussed in a review paper by Wilson et al. (Wilson et al., 2013).

Rupture of AAA is facilitated by the structural degradation of the arterial wall until the mechanical stress acting on the wall exceeds the strength of the wall tissue. It generally occurs in conjunction with the formation of the ILT in the lumen of the AAA bulge. ILT prevalence at rupture is estimated to occur in over 75% of clinically studied AAAs, though the precise role in facilitating rupture remains unclear. However, it is known that ILT presence influences the localized transluminal oxygen diffusion. It has furthermore been suggested that the geometric factors of the ILT may influence its role in AAA rupture (Riveros, 2013). Radially thicker ILT, for example, particularly inhibits oxygen diffusion through the aneurysmal wall, which leads to greater local oxygen loss, also known as hypoxia, more so than with radially thinner ILT. This resulting localized oxygen deprivation is thought to lead to degradation of the arterial wall, causing sufficient conditions for AAA rupture.

In order to maintain the health of the arterial wall, sufficient transluminal oxygen transport is essential. However, there is insufficient evidence produced to date from which definitive conclusions can be drawn towards the precise role of particularly thrombus-mediated limitations to mass transport in AAAs, though several studies have demonstrated the phenomenon of “Hypoxia-Mediated Wall Weakening” and have asserted that the morphology of ILTs can play a key role to directly influence collagen and elastin production, induce hypoxia in the arterial wall, and decrease wall strength (Vorp et al., 1998; Vorp et al., 2001). These investigations outline the potential importance of hypoxia in upsetting the local balance of protein degradation and synthesis in the AAA wall (see a comprehensive review in (Vorp and Geest, 2005)). The observed local hypoxic environment in AAA may therefore lead to a decrease in the overall structural integrity of the wall and its eventual rupture. In particular, Vorp et al. examined the hypothesis that ILT in AAA is associated with local hypoxia of the AAA wall (Vorp et al., 2001).

While hypoxia can detrimentally impact the structure and content of the wall, the mechanical stresses within the wall ultimately mediate its rupture. Most previous studies have analyzed the effect of ILT presence on transmural wall stress in AAA models (Mower et al., 1997; Wang et al., 2002; Di Martino and Vorp, 2003; Li et al., 2008; Bluestein et al., 2009; Haller et al., 2018), where qualitative information is obtained on how ILT influence aneurysm wall stress. Though the presence of ILT can inhibit physiologically healthy transluminal oxygen transport in AAA, these studies have found that from a mechanical vantage, ILT can act as structural enhancement; thus, reducing the risk of aneurysm rupture by bearing some of the arterial mechanical load. In this manner, a few studies investigated the role of geometric factors in rupture assessment of AAA. However, previous studies on the effect of AAA geometrical features have been only focused on statistical association between geometrical asymmetry, wall stress, and rupture; without considering either the ILT region or the oxygen transport within the AAA (Li and Kleinstreuer, 2007; Bhagavan et al., 2018). Yet, there has not been any research to the authors’ awareness on the thrombus-mediated limitations to mass transport in AAAs; particularly towards the gross geometric ILT features which, in addition to regulating mass transport, can later provide information towards AAA structural stability. Including the ILT as a geometric feature and investigating the relation between its morphology and oxygen flow to the aneurysmal wall is the main purpose of our study.

In some previously reported experimental and computational investigations, the effect of blood flow on convective mass transfer of oxygen molecules had been neglected and only the ILT and wall regions had been considered (i.e., (Vorp et al., 1998; Polzer et al., 2012)). In particular, in (Vorp et al., 1998) a uniform oxygen concentration was assumed on the luminal surface to model the oxygen supply from the blood. However, given the advective-diffusive nature of fluid flow in luminal oxygen transport, including the hemodynamic features may provide additional insight, as previously, it has been shown that hemodynamics plays a key role in the development of ILT (Virag et al., 2015), and the blood flow field affects the oxygen transport to the arterial wall particularly in the regions of disturbed flow and reattachment. Therefore, the coupling between blood-side oxygen mass transport to the transport in the wall is necessary to accurately model local oxygen concentrations within the AAA. In (Sun et al., 2009), this coupling between hemodynamics and mass transport for a patient-specific model of AAA is considered. However, using patient-specific models without having knowledge about the variations from patient-to patient in flow rate and physical properties, prohibits a thorough sensitivity analysis of the model’s parameters.

Our previous studies in (Zakerzadeh et al., 2021) and (Zakerzadeh et al., 2020) have thoroughly explored the effect of varying ILT biomechanical parameters such as permeability and oxygen diffusivity in an idealized AAA model. We extended the work by Vorp et al. (Vorp et al., 1998) in a parametric study and implemented a computational framework of coupled blood flow and oxygen transport for the purpose of assessing the effects of different geometrical and physical features including vasa vasorum flow (in the form of varying abluminal oxygen concentration boundary condition), kinematic diffusivities within the AAA tissue, and oxygen consumption in the arterial wall. Moreover, we also analyzed the effect of AAA geometry using four similar AAA models as in (Vorp et al., 1998) and observed that geometry seems to have substantial influence on mass transport, and in particular, that ILT geometry can play a notable or negligible role on oxygen transport depending on its geometry (Zakerzadeh et al., 2021). However, only four different geometries were used which is not enough to make a conclusion.

In the current work, we augment this via parametric modeling of both luminal and ILT geometries with the purpose of systematically investigating the role of changing ILT length and thickness in idealized AAAs, which are themselves parametrically varied. Additionally, in previous studies, the correlation between oxygen diffusion and ILT geometry is not examined in detail as it is in this manuscript. This study is the first attempt to explore the association of different morphological features of ILT with the oxygen delivery to the AAA and the possible wall oxygen deprivation, in a systematic and comprehensive manner.

With the use of physiologically realistic biomechanical simulations of coupled computational fluid flow and mass transport in AAA, we simulate aortic wall oxygen transport and investigate the extent to which ILT attenuates oxygen concentration in the arterial wall. A three-dimensional computational model of AAA containing ILT is constructed which is similar to the previous research by authors presented in (Zakerzadeh et al., 2021). The parametric space is composed of twenty-seven AAA models. Geometrical parameters include AAA bulge diameter, which is related to ILT curvature, ILT thickness, and ILT length. The modeling framework accounts for fluid dynamics in the lumen and oxygen transport in the lumen, thrombus, and arterial wall. Blood flow in the lumen is modeled using Navier-Stokes equations and then coupled with advection-diffusion-reaction equations that model the transport of biomolecules in AAA and their interaction with the arterial wall living tissue. This coupled blood flow-oxygen transport model is utilized in order to analyze the influence that ILT size has on oxygen concentration attenuation in the arterial wall. Computational results on velocity fields inside the lumen and oxygen concentrations inside the ILT and wall are obtained. Twenty-seven different geometries are simulated, and concentration measures at various locations within the tissue and along the boundaries are presented and compared.

The manuscript is organized as follows. In *Materials and Methods*, we describe the tools that we use to perform AAA simulations: *Geometry Variations and Parametric Space* introduces the geometrical space, provides the geometrical representation of models that we use for each simulation, and explains how the twenty-seven cases are created. *Governing Equations and Formulation* and *Boundary Conditions and Physical Parameters* cover the governing equations for the representation of the blood flow and oxygen transport within AAA and provide the mathematical model parameters and boundary conditions. In *Solver details*, we summarize the numerical simulation techniques. In *Results*, we explore the numerical results and compare them for simulated AAA cases. Finally, the discussion of the results, conclusions and future directions are presented in *Discussion*.

## Materials and Methods

**FIGURE 1:**
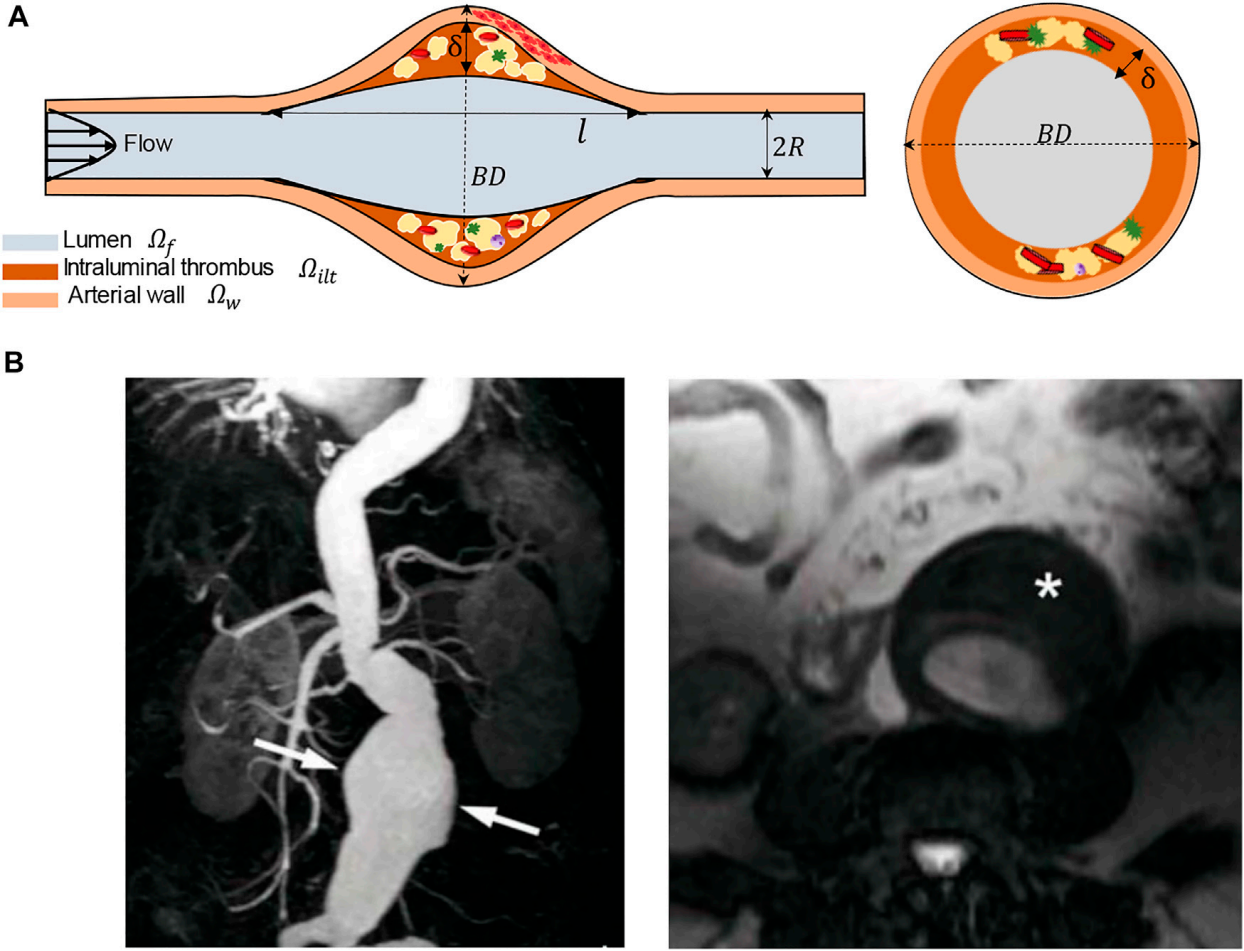
**(A)** Schematic of the idealized, three-dimensional axially symmetric model of an AAA, where *BD* represents the bulge diameter, l denotes ILT length, and δ is the ILT thickness. **(B)** Left: coronal plane view of a magnetic resonance angiography (MRA) of a clinical infrarenal AAA. Extensive dilatation (arrows) and renal arteries on both sides can be observed. The renal arteries in MRA image are not included in the computational model. Right: the image demonstrates aneurysm formation and thrombus-covered region (asterisk). MRA images are taken from (Douek, 2005).

In this section, we summarize the steps to achieve a realistic simulation of the fluid flow and oxygen transport within AAA. We have constructed three-dimensional CAD models of an axisymmetric AAA containing an ILT. The maximum diameter of the AAA occurs at halfway along the vessel length and is called the bulge diameter (*BD*). A schematic of the axial and transverse cross-sections of the model is presented in Figure 1 and Table 1 contains the geometric parameters used to create the model and summarizes the ranges of ILT thickness and ILT length examined in our study.

**TABLE 1:** Geometric modelling parameters and their values in idealized lesions.

Geometric parameter	Value	Geometric parameter	Value
R (cm)	1 Hirsch, (2006)	Short ILT length (cm)	4
Length (cm)	24 Scotti et al. (2005)	Medium ILT length (cm)	6 Wolf et al. (1994)
Wall thickness (cm)	0.1	Long ILT length (cm)	8
Small-sized AAA bulge diameter (cm)	4.2 Bhagavan et al. (2018)	Thin ILT thickness (cm)	0.4 Vorp et al. (2001)
Moderate-sized AAA bulge diameter (cm)	5 Zhu et al. (2020)	Medium ILT thickness (cm)	0.8 Wang et al. (2001)
Large-sized AAA bulge diameter (cm)	7 Vorp et al. (1998); Cnotliwy, (2010)	Thick ILT thickness (cm)	1 Zhu et al. (2020)

### Geometry Variations and Parametric Space

**FIGURE 2:**
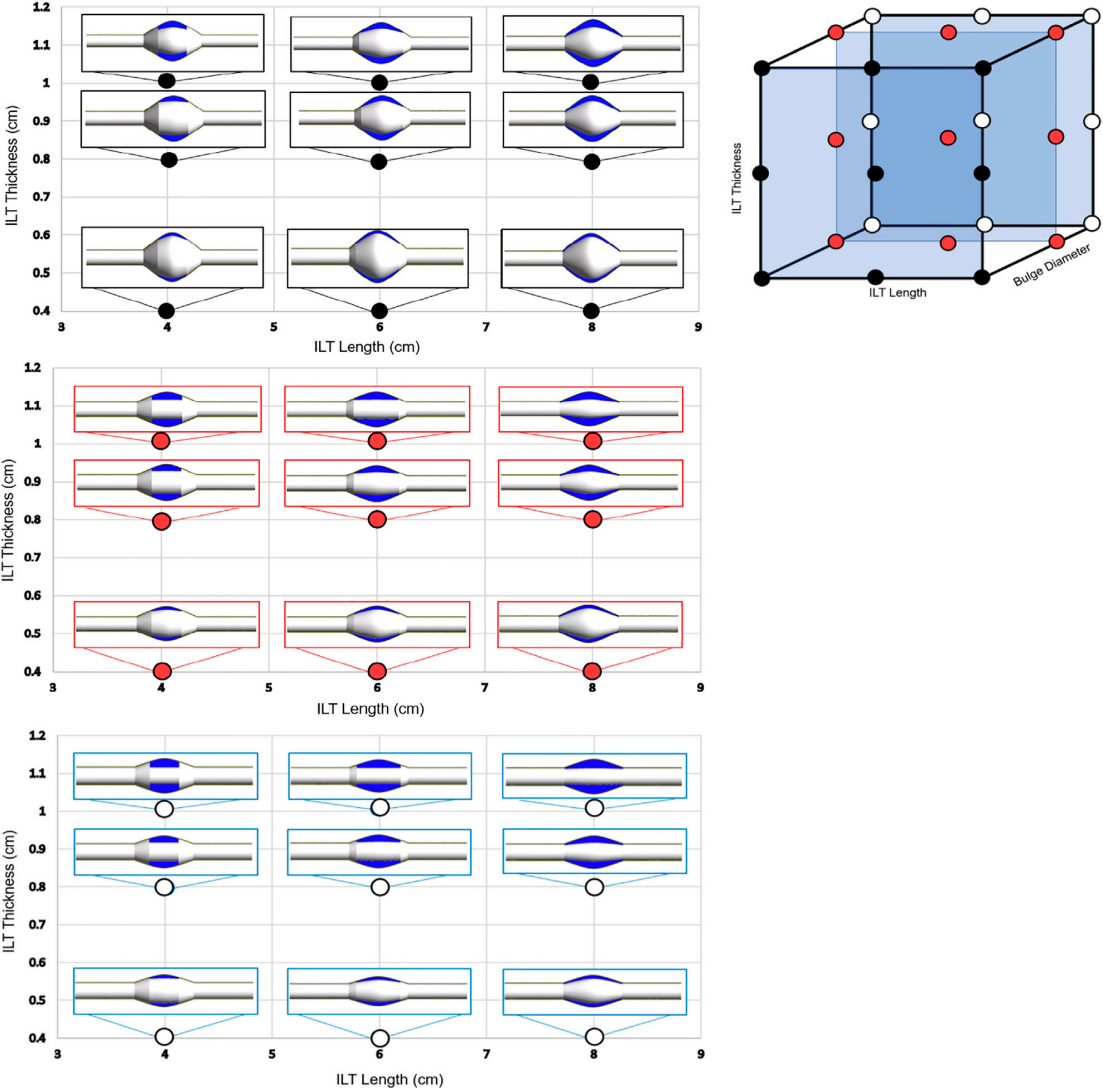
The 3-dimensional visualization of the parametric space is shown (top-right), where each point represents one of the twenty-seven AAA geometries used in this study. Each case is characterized by a variation in ILT length, bulge diameter, and ILT thickness identified on one of the three axes, respectively. The cases are divided into three groups of nine AAA models based on bulge diameter, denoted by black (*BD* = 7 cm), red (*BD* = 5.2 cm), and white (*BD* = 4.2 cm) points on the 3D parametric space cube. The three 2D plots (left) illustrate the anatomic variations on three planes of constant *BD*, and each plane shows combined variations in thickness and length of the ILT lesion. Individual cases were designed to have ILT length of 4 cm, 6 cm, or 8 cm, and an ILT thickness of 0.4, 0.8, or 1 cm. These cases will serve as the basis of comparison for differences oxygen concentration among the simulations.

The parametric study space consists of twenty-seven idealized geometries. Each AAA case has three bodies: the wall, the blood, and the ILT. AAA models were designed in the commercially available Fusion 360 CAD software, and preprocessed and meshed using the ANSYS SpaceClaim, a geometry component in ANSYS Workbench (version 20.2, ANSYS Inc., Canonsburg, PA, USA). For each bulge diameter (*BD*) used, the wall was sketched and saved as a framework. Using this framework, each ILT was sketched to desired length ( l ) and thickness ( δ ). The wall and ILT sketches were then revolved around the central axis to become independent 3-D bodies. Finally, the blood body was created by filling the extra area within the vessel. This process was repeated for each of the twenty-seven AAA models. A visualization of these modeling parameters and geometric measurements can be observed in Figure 2. The AAA and ILT dimensions are agreeable with patient-specific morphological data (Hans et al., 2005) and are listed in Table 1. For each case, the AAA domain is defined as 24 cm long, with arterial wall thickness held constant at 0.1 cm. The blood vessel is defined with a radius of *R* = 1 cm at the inlet and outlet of each case, with larger bulge protrusions within the middle of the arterial domain as specified in Figure 1.

Bulge diameters of 4.2, 5, and 7 cm are assumed for each set of simulations, These *BD* values are in the range of baseline diameters for demographic data of patients’ aneurysm size to simulate small, medium, and large aneurysm (see (Wolf et al., 1994; Cnotliwy, 2010; Zhu et al., 2020)). Bulge diameter is kept constant for each aneurysm size, and the ILT thickness and ILT length are varied to create three groups of nine AAA cases (Figure 2). ILT thickness and length vary with ranges of δ = 0.4, 0.8, 1 cm and l = 4, 6, 8 cm, respectively. Thus, the analysis accounts for the combination of “short/medium/long” and “thin/medium/thick” ILTs in small, medium, and large aneurysms. The thickness of the ILT region is chosen according to relevant clinical data (Cnotliwy, 2010; Xenos et al., 2015), and the range for the ILT length is calculated from patient ILT volume and ILT thickness demographics measurements (Ghulam et al., 2018). An illustration of the parameter space and AAA geometries spanning the full ranges of the three explicit parameters *BD*, δ and l are shown in Figure 2. The 3-D parameter space is divided into three 2-D plots that partition cases by *BD* values (black corresponds to *BD* = 7 cm, red represents *BD* = 5.2 cm, and white signifies *BD* = 4.2 cm). In each plotted image, AAA lumen is colored white, and ILT domain is depicted in blue. Variations in ILT length (in cm) are plotted against ILT thickness (in cm) in each plot.

### Governing Equations and Formulation

Our mathematical model consists of three domains: luminal blood, the ILT, and the arterial wall. Since the aorta is a large artery, blood flow in the lumen is assumed to behave as a Newtonian viscous fluid. Hence, the steady, incompressible, laminar blood flow inside the artery channel is described by the Navier–Stokes equations:

$$\rho_{f}\left(U_{f}.\;\nabla U_{f}\right)=\nabla .\sigma_{f}\;in\;\Omega_{f}$$ (1)

$$\nabla .U_{f}=0\;in\;\Omega_{f}$$ (2)

Here $U_{f}$ and $\rho_{f}$ stand for fluid velocity vector field in the lumen and blood density, respectively, and $\sigma_{f}=-p_{f}I+2\mu_{f}D\left(U_{f}\right)$ is the fluid Cauchy stress tensor where $p_{f}$ is fluid pressure, $\mu_{f}$ denotes blood dynamic viscosity, and the symmetric part of the fluid velocity gradient is defined as $D\left(U_{f}\right)=\frac{1}{2}\left(\nabla U_{f}+\nabla U_{f}^{T}\right)$ . It should be noted that the subscripts f , w and ilt indicate the blood flow in the lumen, AAA wall and ILT medium respectively.

Oxygen transfer in the lumen is coupled with the blood flow and governed by the convection–diffusion equation as follows:

$$\nabla \cdot \left(-D_{f}\nabla C_{f}+U_{f}C_{f}\right)=0\;in\;\Omega_{f}$$ (3)

The symbol $C_{f}$ denotes the oxygen concentration in the lumen and $D_{f}$ is the diffusion coefficient for oxygen in blood. The effect of oxygen binding to hemoglobin within the AAA lumen is neglected, as our focus is confined to the relative differences in oxygen transport across the arterial wall.

Oxygen transport in the AAA wall is modeled by the diffusion-reaction equation and therefore we have:

$$\nabla \cdot \left(-D_{w}\nabla C_{w}\right)=rC_{w}\;\;in\;\Omega_{w}$$ (4)

where $C_{w}$ is the oxygen concentration in the AAA wall, $D_{w}$ is the oxygen diffusivity through the AAA wall, and $rC_{w}$ is the reaction term which accounts for the metabolite consumption of oxygen in cell metabolism. In particular, we introduce the consumption rate S , when the consumption of oxygen is assumed to be linearly proportional to the cell availability, namely $S=rC_{w}$ , and r is defined as the oxygen reaction rate constant (Iannetti et al., 2016).

The ILT is assumed permeable to the transport of oxygen molecules through diffusion via the canaliculi network, without any smooth muscle cells to consume oxygen. Therefore, no reaction term is considered in the thrombus and the oxygen transfer in the ILT can be modeled by the diffusion equation, given by:

$$\nabla \cdot \left(-D_{ilt}\nabla C_{ilt}\right)=0\;\;in\;\Omega_{ilt}$$ (5)

where $C_{ilt}$ is oxygen concentration in the ILT, and $D_{ilt}$ is the oxygen diffusivity through the ILT.

It should be noted that although ILT has a porous structure arising from its highly porous canalicular network (Adolph et al., 1997), neglecting the contribution of convective oxygen transport due to interstitial fluid flow (i.e. the movement of the fluid through the extracellular matrix of the tissue) has been justified in (Polzer and Bursa, 2010; Polzer et al., 2011) where the stress fields in the aneurysmal wall changed negligibly when a poroelastic model for ILT was used. Similar conclusions were obtained in (Ayyalasomayajula et al., 2010) where simulations were performed in order to compare poroelastic and impermeable ILT models, and the ILT permeability was found to have minimal effect on interstitial velocities that have been associated with driving oxygen transport in the ILT and arterial wall. We previously explored the effect of porous ILT on oxygen transport and our findings suggest that the effect of porosity of ILT on oxygen transport within AAA is small for the reported physiological range of ILT permeability (Zakerzadeh et al., 2020).

In the next section, we summarize the parameters and boundary conditions.

### Boundary Conditions and Physical Parameters

In this section, we summarize the main parameters and boundary conditions of the flow and mass transport model. At the inlet of the artery, we considered a fully developed and unidirectional velocity profile. The flowrate is chosen to match physiological Reynolds numbers and represents the flow in the abdominal aorta under the resting condition (Fraser et al., 2008). Therefore, the following parabolic velocity profile for the laminar flow taken from (Caputo et al., 2013) is specified at the inlet:

$$U_{f}^{in}\left(r\right)=2U_{med}\;\left(1-{\left(\frac{r}{R}\right)}^{2}\right)n\;\;on\;\Gamma_{f}^{in}\;$$ (6)

where ***n*** denotes the inward normal unit vector at the inlet surface, R is the radius of the vessel, and we define $r=\sqrt{y^{2}+z^{2}}$ at the inlet. The correspondent mean velocity is obtained as $U_{med}$ = 0.235 m/s from the measurements of the flowrate reported by (Fraser et al., 2008). The fluid is assumed to have density of $\rho_{f}$ =1,050 kg/m^3^ and viscosity of $\mu_{f}$ = 0.0035 kg/m.s, representing human blood properties. The Reynolds number of 1,100 is obtained based on the inlet lumen diameter and the inlet flow condition, which is within the realistic range for Reynolds numbers in abdominal aorta at rest (Ku, 1997). The blood oxygen partial pressure is considered a uniform 100 mmHg (Applegate et al., 2016), corresponding to the oxygen saturation of 97.7% (Severinghaus, 1979). Therefore, the oxygen concentration of $C_{f}^{in}$ =5.12 × 10^−3^ kg/m^3^ at the lumen inlet $\Gamma_{f}^{in}$ is obtained by using the inlet partial pressure and molar mass of oxygen. At the lumen outlet $\Gamma_{f}^{out}$ , the reference pressure value was prescribed, which can be arbitrarily set for incompressible flow. On the exterior of the arterial wall, the influence of the vasa vasorum is modelled by imposing the partial pressure of oxygen. Experimental studies showed that the level of adventitial oxygen tension is about one half of the oxygen tension in blood (Buerk and Goldstick, 1982; Buerk and Goldstick, 1986); therefore the abluminal wall partial pressure was fixed at 50 mmHg to model the oxygen transported by the vasa vasorum (Kemmerling and Peattie, 2018). At the cross-sectional areas of the wall representing the ends of the vascular domain a zero-flux condition on the surface normal direction is prescribed. It should be noted that an adventitial oxygen concentration of 50 mmHg, that has been used for the baseline case and previous computational models of AAA (Sun et al., 2009), corresponds to a healthy infrarenal aorta (Buerk and Goldstick, 1986). However, in the case of AAA it is expected that this value decreases significantly, since the vasa vasorum has been observed to be stenotic in both small and large AAA with the tissue being ischemic and hypoxic (Tanaka, 2015).

Conservative, interface oxygen flux conditions are employed at the ILT-wall ( $\Gamma_{ilt-w}$ ) and ILT-lumen ( $\Gamma_{f-ilt}$ ) interfaces, and a continuity condition was assumed between the boundaries. Moreover, the no-slip condition is defined at the interface between the lumen and wall ( $\Gamma_{f-w}$ ), and the lumen and ILT. Therefore, the model is complemented by the following concentration interface conditions:

$$\;\;C_{f}=C_{w}\;\;on\;\;\Gamma_{f-w}\;\;\;C_{f}=C_{ilt}\;\;on\;\Gamma_{f-ilt}\;\;\;C_{ilt}=C_{w}\;on\;\Gamma_{ilt-w}\;\;\;$$ (7)

The transport equation in the fluid model describes oxygen transport with a kinematic diffusivity of $D_{f\;}$ =1.6 × 10^−9^ m^2^/s (Rappitsch and Perktold, 1996). The values for AAA wall and ILT diffusivity are taken from previous studies, where $D_{ilt}$ =1.34 × 10^−9^ m^2^/s and $D_{w}\;$ =1.08 × 10^−9^ m^2^/s are the diffusivities of oxygen through the ILT and wall respectively. The oxygen reaction rate is assumed to be r  =8.4 × 10^−3^ s^−1^ under the assumption that the volume flux of oxygen is completely consumed by the smooth muscle cells. A summary of all model parameters is provided in Table 2.

**TABLE 2:** Physical parameters and material properties for AAA model domains.

Simulation parameter	Blood	Aneurysmal wall	ILT
Thermodynamic state	Incompressible fluid	Impermeable solid	Impermeable solid
Oxygen diffusivity (m^2^/s)	1.6 × 10^−9^ Rappitsch and Perktold, (1996)	1.08 × 10^−9^ Moore and Ethier, (1997)	1.34 × 10^−9^ Sun et al. (2009)
Density (kg/m^3^)	1,060	1,000	1,000
Dynamic viscosity (kg/m.s)	0.0035		
Reaction rate (s^−1^)		8.4 × 10^−3^ Caputo et al. (2013)	
Reynolds number	1,100 Ku, (1997)		
Inlet oxygen concentration (kg/m^3^)	5.12 × 10^−3^ Vorp et al. (1998)		
Vasa vasorum oxygen concentration (kg/m^3^)	2.06 × 10^−3^ Kemmerling and Peattie, (2018)		

### Solver Details

Twenty-seven fully coupled hemodynamic and oxygen transport simulations by obtaining the solutions of the Navier-Stokes equations plus diffusion-reaction equations were performed. The model was implemented in commercial code ANSYS CFX Workbench software (version 20.2, ANSYS Inc., Canonsburg, PA, USA) to carry out the fluid dynamic and mass transport simulations. Numerical simulations were performed to simulate blood flow coupled with the oxygen transport in the bloodstream and oxygen diffusion in the wall and ILT regions. The spatial discretization consists of a cell based finite volume method. A fully coupled strategy has been adopted, namely all the equations are solved simultaneously through a monolithic linear system that embraces all the degrees of freedom. The Laplace operator in the fluid momentum and oxygen transport equations is approximated by a centered scheme, while the convective terms have been discretized by means of an upwind method. The convective term in the Navier-Stokes equations is linearized by the Picard iterations (“ANSYS CFX-Solver Theory Guide”, ANSYS Inc., 2010). The pressure variable in the Navier-Stokes equations is evaluated at the same nodes of the velocity field. The system is then solved using an algebraic multigrid method exploiting incomplete LU factorization as smoother. Convergence criteria were set to $10^{-5}$ for the normalized residuals of the global linear system of equations. Numerical simulations have been performed on parallel CPUs using a 5-Core Intel CPU, 32 GB RAM at Duquesne University. The problem was solved using the MPI parallel solver. Each simulation computes the steady-state solution using four processor cores. To obtain a reasonable resolution of the solution based on the assumed value of the Reynolds number of 1,100, we used about 1.5 million tetrahedral elements to discretize the domain representing AAA. The mesh resolutions are identical to those reported in Zakerzadeh et al. (2021). The cited study also documents a systematic mesh convergence testing which is similar to that used in the computations of the current model.

## Results

In this section, we investigate the dependence of oxygen distribution within a AAA domain on the variation of the ILT and AAA geometrical features including ILT thickness, ILT length and AAA bulge diameter that is directly linked to the ILT curvature. In particular, our computational solver provides a spatial prediction of oxygen concentration in the ILT and arterial tissue, as well as hemodynamic measures inside the lumen. Twenty-seven different geometries created in *Geometry variations and parametric space* are used, and the oxygen concentration profiles at different locations are presented and compared. These geometries are referred to by case (l,δ) for each *BD* values, where l is ILT length and δ is ILT thickness.

**FIGURE 3:**
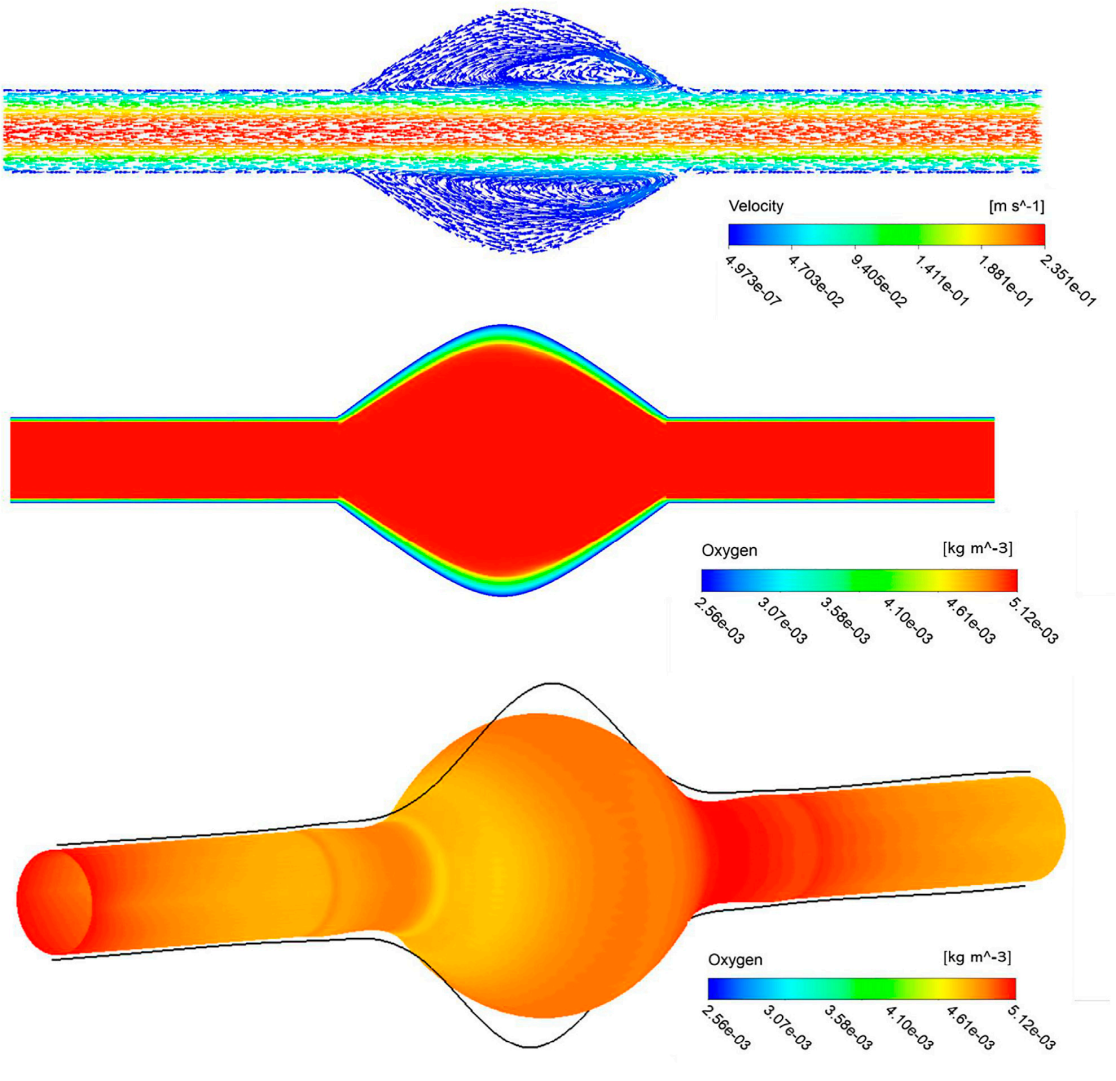
Stream traces colored by the velocity in the lumen (top), arrowheads over the streamlines convey the direction of flow. Profile of oxygen concentration in AAA including lumen, ILT and wall domains, on a plane that cuts the domain in half (middle), distribution is marked with the highest concentration in the lumen and lowest in the AAA wall. The contour of oxygen concentrations on the luminal surface (bottom).

We first present the results of a baseline simulation to investigate hemodynamics and mass transport results. In the top panel of Figure 3, visualization of blood streamlines colored by the velocity magnitude, on the plane that cuts the AAA in half is provided. The pattern of streamlines in the blood indicates the distribution of the flow within the lumen. It is seen that blood flow in the AAA decelerates due to the enlarged diameter of the vessel and the streamlines show recirculation zones characterized by helical flow pattern in the bulge of the lumen. Additionally, the oxygen concentration distribution contour inside the whole domain that contains lumen, ILT, and wall, is shown in the middle panel of Figure 3. The lumen appears red, indicative of oxygen-rich blood. Moving toward the exterior of the artery, this high oxygen concentration inside the lumen that is spatially uniform, diminishes rapidly with distance inside the ILT and arterial wall. Therefore, the highest oxygen concentration is found in the lumen and the lowest is in the wall as expected. We observe more reduced oxygenation of the aortic wall in the presence of ILT which is in the middle of the lumen. This oxygen deprivation is most noticeable at the maximum diameter of the AAA, *BD*, that occurs at halfway along the length of the lumen and has the highest thickness of ILT (see Figure 1 for a schematic illustration of the model). The oxygen concentration is higher at the straight portion of the vessel that thrombus does not exist. This observation is in line with previous conclusions in (Vorp et al., 2001). Furthermore, the bottom panel of Figure 3 illustrates contour of oxygen concentrations on the luminal surface, defined as the interface between the lumen and ILT and between the lumen and the wall. This oxygen contour clearly shows that the concentration is not uniform at the blood-tissue interface and changes between 5.12 × 10^−3^ kg/m^3^ to 4.43 × 10^−3^ kg/m^3^. The observation highlights the influence of hemodynamics to compute a more realistic oxygen concentration on the luminal surface and use that to solve the oxygen diffusion within the ILT and wall tissue.

To quantify the sensitivity of oxygen flow measures to the chosen space of geometric values provided in Table 1 and Figure 2, various plots are prepared and arranged that illustrate the results on different points, lines, and surfaces, such as within the ILT tissue, on the abluminal ILT layer, and on the outermost layer of the arterial wall that is referred to as tunica adventitia.

**FIGURE 4:**
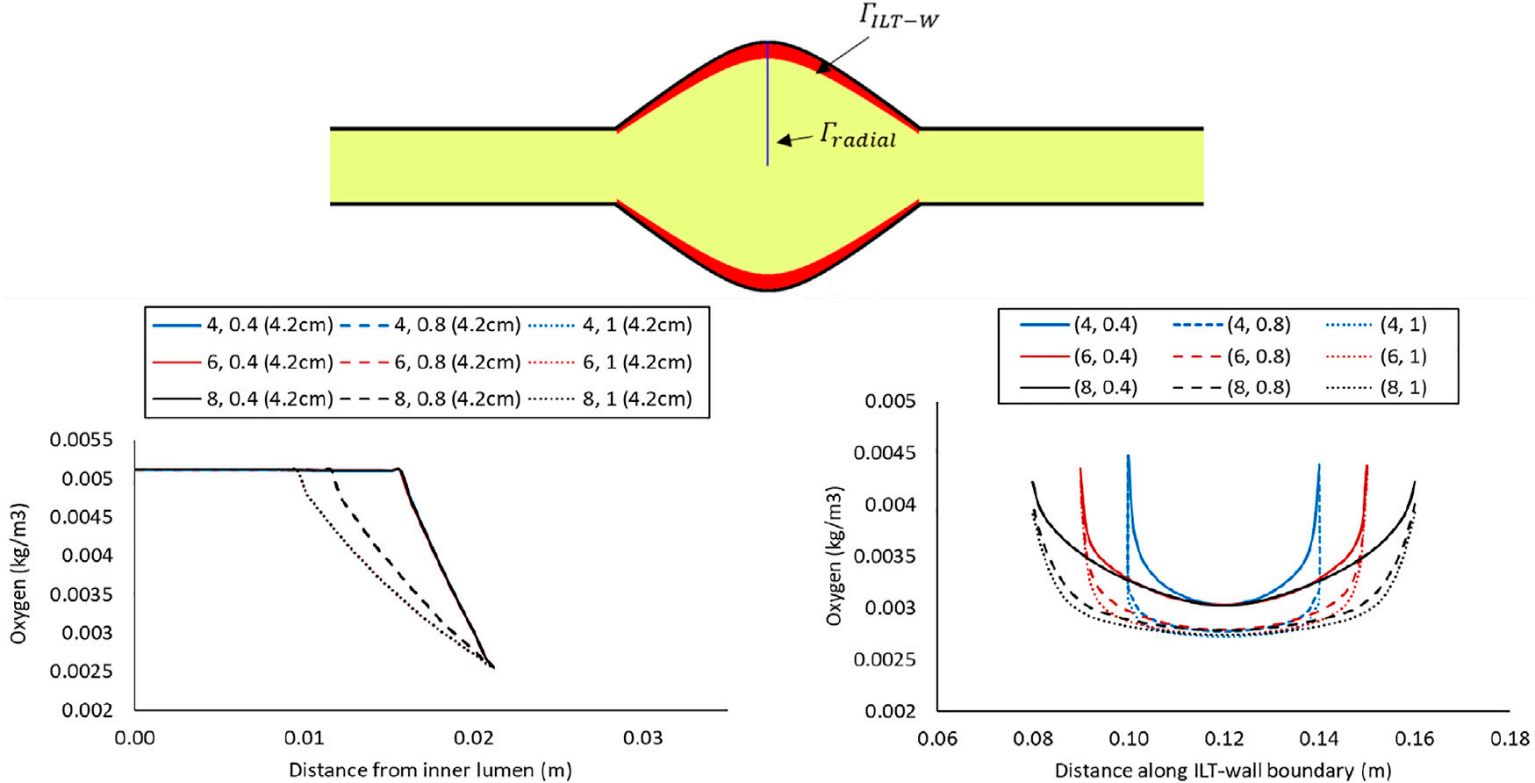
Comparison of the oxygen concentration along a radial path line at halfway along the length of the lumen in the cases with *BD*= 4.2 cm and varying ILT length ( l ) and ILT thickness ( δ ) denoted by case (l,δ) (left), and comparison of the oxygen concentration along the axial length on the interior interface of the arterial wall in the same cases (right). In both plots, the blue, red, and black lines represent the short, medium, and long ILT cases information respectively; and solid, dashed, and dotted lines represent thin, moderate and thick ILT cases respectively. All nine cases are shown in Figure 2. At the top, these path lines are shown with blue line, $\Gamma_{radial}$ for the left plot and $\Gamma_{ILT-W}$ for the right plot. The $\Gamma_{ILT-W}$ represents the interface between the ILT (red region) and wall domain (black region).

**FIGURE 5:**
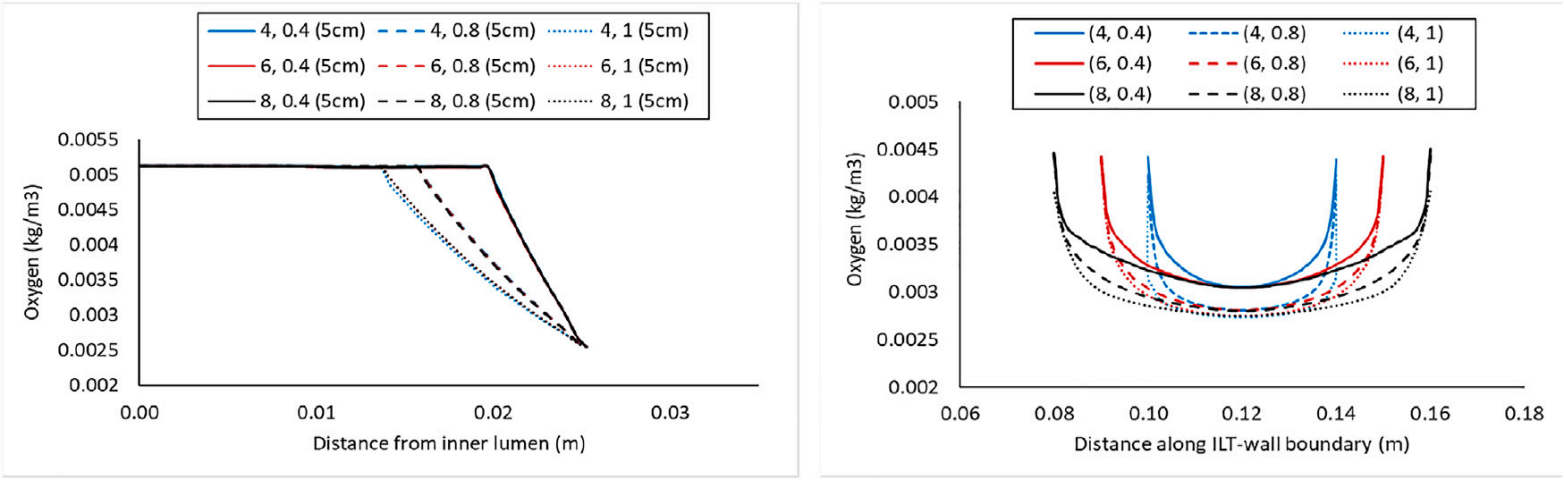
Comparison of the oxygen concentration along a radial path line at halfway along the length of the lumen in the cases with *BD*= 5 cm and varying ILT length ( l ) and ILT thickness ( δ ) denoted by case (l,δ) (left), and comparison of the oxygen concentration along the axial length on the interior interface of the arterial wall in the same cases (right). In both plots, the blue, red, and black lines represent the short, medium, and long ILT cases information respectively; and solid, dashed, and dotted lines represent thin, moderate, and thick ILT cases respectively. Cases are presented in Figure 2. Path lines are demonstrated in Figure 4.

**FIGURE 6:**
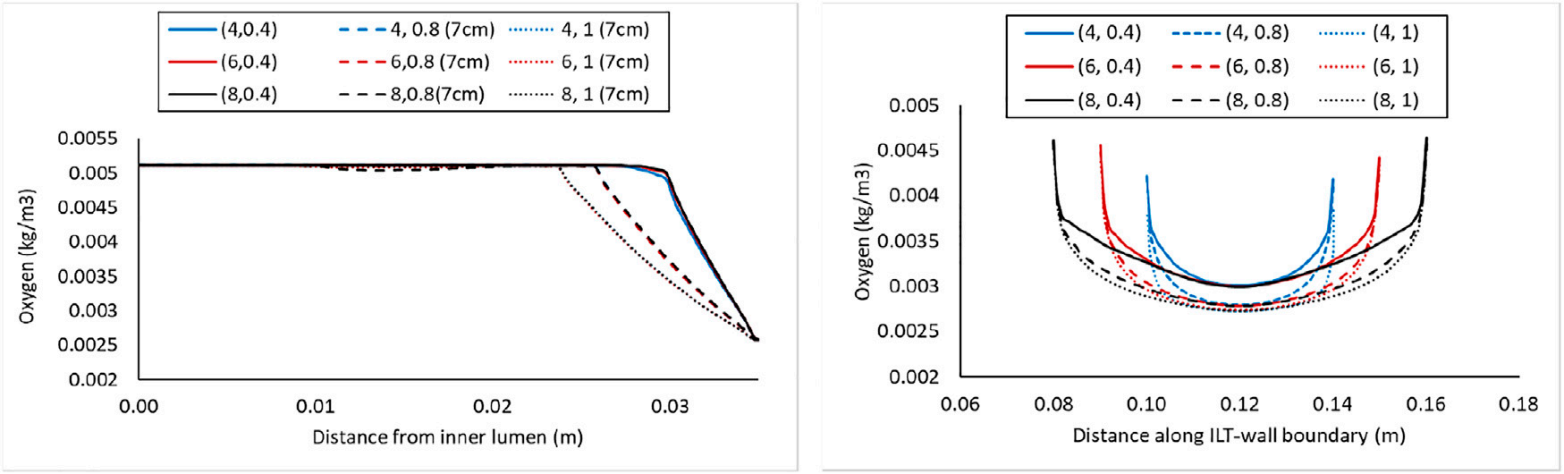
Comparison of the oxygen concentration along a radial path line at halfway along the length of the lumen in the cases with *BD*= 7 cm and varying ILT length ( l ) and ILT thickness ( δ ) denoted by case (l,δ) (left), and comparison of the oxygen concentration along the axial length on the interior interface of the arterial wall in the same cases (right). In both plots, the blue, red, and black lines represent the short, medium, and long ILT cases information respectively; and solid, dashed, and dotted lines represent thin, moderate, and thick ILT cases respectively. Cases are presented in Figure 2. Path lines are demonstrated in Figure 4.

The lines plots in Figures 4–6 depict oxygen concentration fluctuation from lumen to outer wall surface as well as the change in oxygen distribution on inner wall surface on the boundary between ILT and wall. Oxygen concentration data for individual cases in each plot is differentiated by color (blue, red, or black color) for the ILT length and line type (solid, dashed, dotted) for ILT thickness, respectively. In the left panel of Figure 4, the concentrations for different case (l,δ) with *BD*= 4.2 cm and varying ILT length ( l ) and ILT thickness ( δ ) are shown along a vertical line that starts at the center of the domain. The path line is identified by blue color at the schematic representation in the top of the aforementioned figure. We observe that the concentration is constant for all nine cases in the lumen and it starts to decrease exponentially once we reach the ILT interface. For all the cases, the concentration eventually reaches the value estimated by vasa vasorum oxygen supply at the tunica adventitia. Figure 4 (left panel), indicates that oxygen concentration value along a radial line in the middle of AAA domain is largely affected by ILT thickness, although it is not affected by ILT length as expected. Furthermore, thinner ILTs resulted in a more sudden change in oxygen concentration from blood to the tunica adventitia that happens over a shorter length of depletion, while thicker ILTs cause a more gradual oxygen decrease that starts closer to the center of the lumen.

In the right panel of Figure 4, the oxygen concentration on the abluminal layer of ILT is shown for the same cases. This plot is obtained along the length of the AAA, measured on the inner wall surface that is identified by an arrow and the green line at the top of the plot. Wall oxygen concentration is captured in the middle portion of the geometry where the ILT is located. We are particularly interested in this area due to the presence of ILT. We observe that oxygen concentration on the inner interface of the aneurysmal wall has the lower values for the cases with thicker ILT tissue (Figure 4, right panel). The lowest concentration is captured at the ILT location where the artery has the maximum bulge. In particular, cases with the similar line pattern, which represents the same ILT thickness, result in the similar minimum value for oxygen concentration on inner wall surface. The oxygen diffusion decreases as ILT thickness increases for all cases. Therefore, the dotted and dashed lines (1 and 0.8 cm ILT thickness values, respectively) are associated with lower oxygen concentration compared to the solid lines (0.4 cm ILT thickness) for all the values of ILT length. Moreover, the results indicate that the longer the ILT is, the more even the oxygen distribution is along the ILT abluminal layer (i.e., ILT-wall interface). With shorter ILTs, the oxygen concentration fluctuates at a higher rate along the length of this region, while a more uniform concentration for longer ILT is observed.

Concentration values along the radial path line for all nine case (l,δ) with *BD*=5 cm is plotted in the left panel of Figure 5, and the oxygen concentrations on the inner wall surface along the length of the AAA are shown in the right panel of Figure 5. The path lines are the same as the one identified at the top of Figure 4. Finally, similar plots for nine cases (l,δ) with *BD*=7 cm are provided in Figure 6. As bulge diameter increased from Figure 4 to Figure 6, we observe that oxygen reduction at the ILT domain appears at a further distance from the center of the lumen.

**FIGURE 7:**
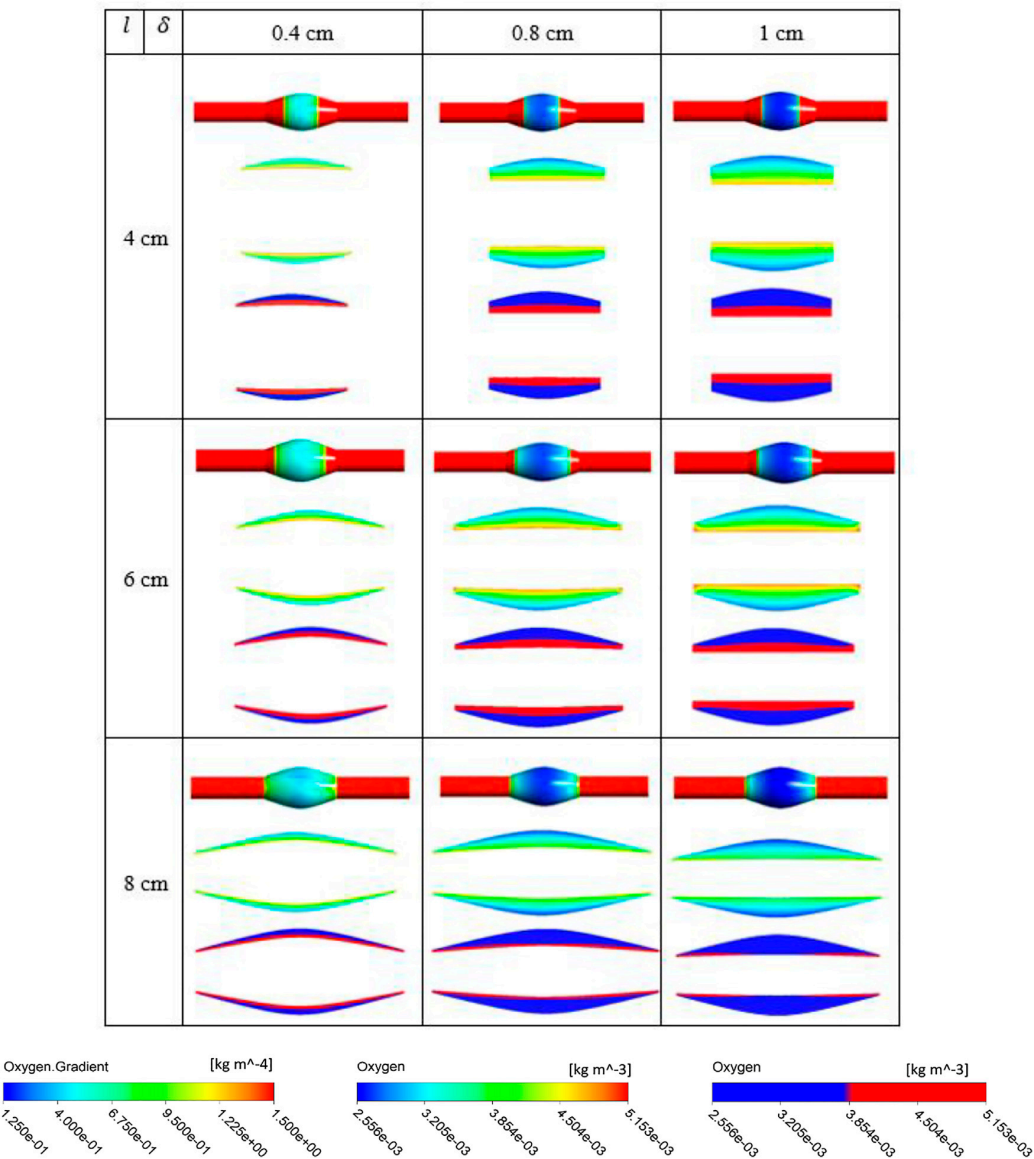
Results of different combination of design variables ILT length ( l ) and ILT thickness ( δ ), with bulge diameter being fixed as *BD*= 4.2 cm. Each cell corresponds to the results for one case of the nine different AAA geometries provided in Figure 2. In each cell, three oxygen measures are plotted from top to bottom: contour of oxygen flux on the abluminal surface of the arterial wall for all analyzed cases on the same scale, contour plot of oxygen concentration in ILT on the same scale, and finally oxygen value using a two-bands color scale ranging from abluminal oxygen concentration 2.56 × 10^−3^ kg/m^3^ (blue) to ≥75% $C_{f}^{in}$ kg/m^3^ (red). Scales on the bottom are used for displaying results.

**FIGURE 8:**
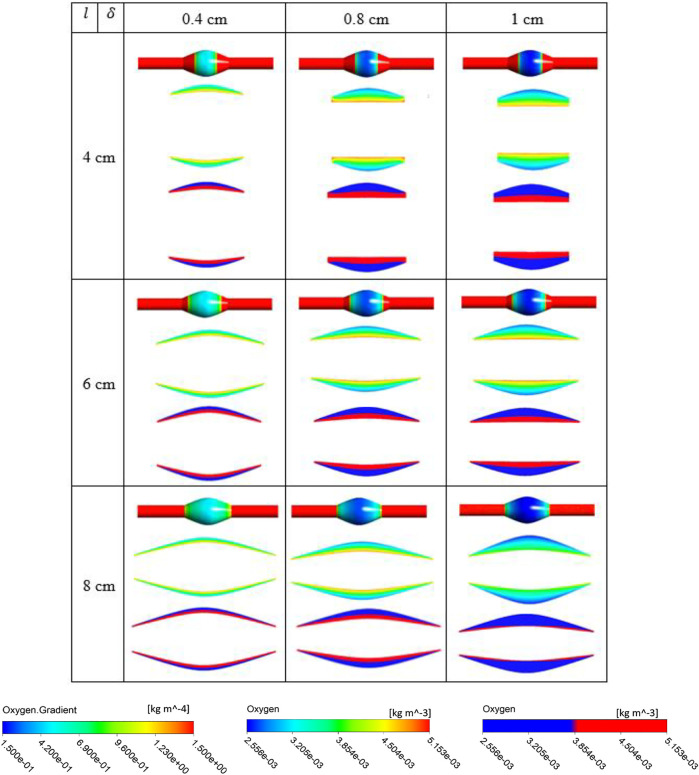
Results of different combination of design variables ILT length ( l ) and ILT thickness ( δ ), with bulge diameter being fixed as *BD*= 5 cm. Each cell corresponds to the results for one case of the nine different AAA geometries provided in Figure 2. In each cell, three oxygen measures are plotted from top to bottom: contour of oxygen flux on the abluminal surface of the arterial wall for all analyzed cases on the same scale, contour plot of oxygen concentration in ILT on the same scale, and finally oxygen value using a two-bands color scale ranging from abluminal oxygen concentration 2.56 × 10^−3^ kg/m^3^ (blue) to ≥75% $C_{f}^{in}$ kg/m^3^ (red). Scales on the bottom are used for displaying results.

**FIGURE 9:**
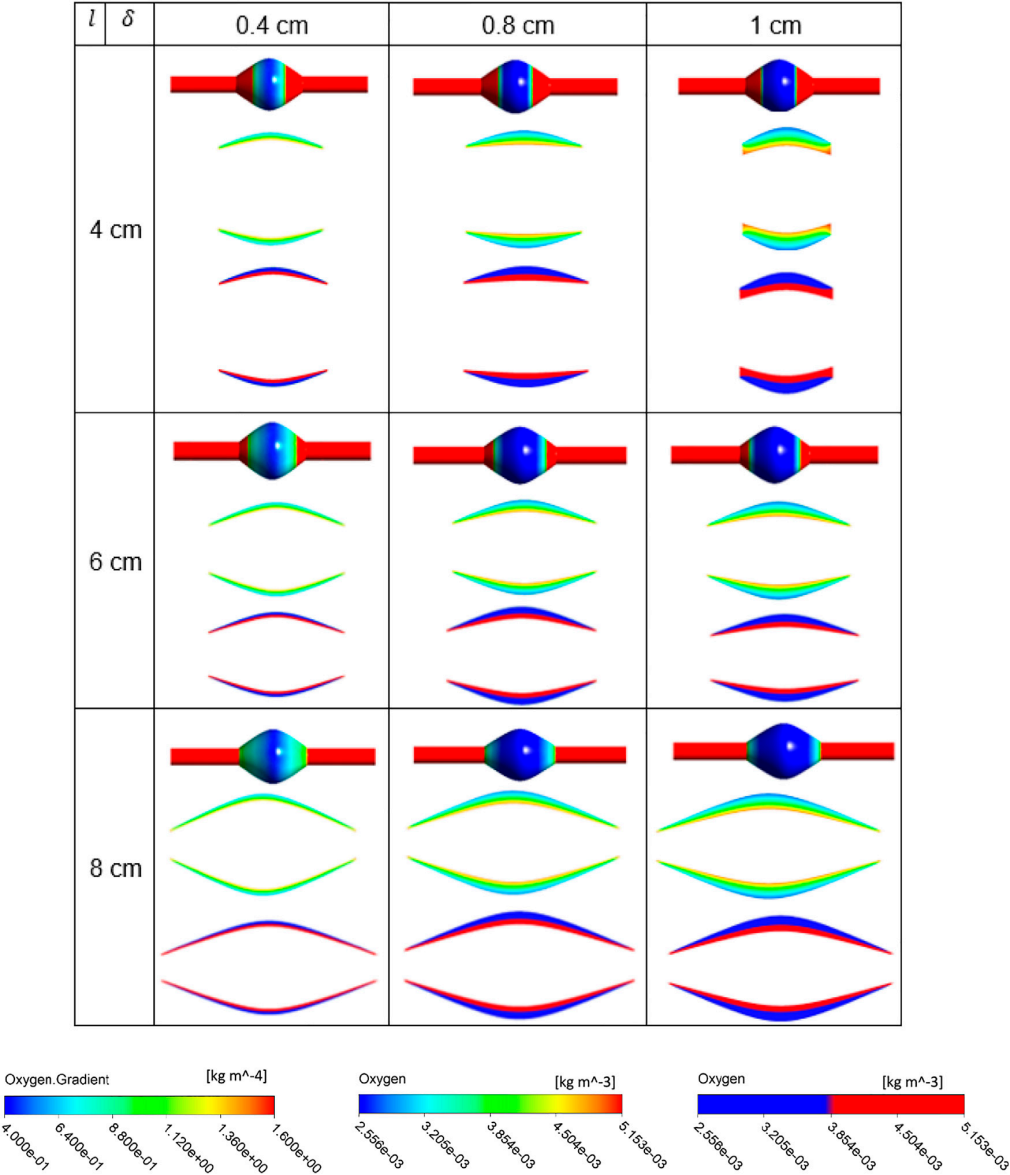
Results of different combination of design variables ILT length ( l ) and ILT thickness ( δ ), with bulge diameter being fixed as *BD*= 7 cm. Each cell corresponds to the results for one case of the nine different AAA geometries provided in Figure 2. In each cell, three oxygen measures are plotted from top to bottom: contour of oxygen flux on the abluminal surface of the arterial wall for all analyzed cases on the same scale, contour plot of oxygen concentration in ILT on the same scale, and finally oxygen value using a two-bands color scale ranging from abluminal oxygen concentration 2.56 × 10^−3^ kg/m^3^ (blue) to ≥75% $C_{f}^{in}$ kg/m^3^ (red). Scales on the bottom are used for displaying results.

The simulation results for the variation of the ILT geometry and quantities of interest for each case are reported in Figure 7 to Figure 9. The quantities include oxygen flux profile on the tunica adventitia layer, oxygen concentration profile within the ILT, as well as a two-level contour plot that is used as a measure to visualize the reduction in oxygen concentration inside the ILT tissue. More precisely, each grid of Figure 7–9, contains the simulations result for these quantities in *BD*= 4.2, 5, and 7 cm respectively. First, the contour of oxygen flux distribution on the aneurysmal wall for each case is shown, followed by the oxygen concentration contour within the cross section of the ILT tissue. Finally, the two-level concentration contour within ILT region is provided. The map has a “cutoff” value to signify low oxygen levels and is colored in red for areas that have an oxygen concentration above a particular value and blue in areas when the oxygen concentration falls below that value. Mainly, display of a two-bands color scale ranges from abluminal oxygen concentration of 2.56 × 10^−3^ kg/m^3^ from vasa vasorum supply, defined by the blue color, to 75% of inlet oxygen concentration $C_{f}^{in}$ identified by the red color. The “inflection point” is reached when the concentration values drop below 75% of the inlet concentration. This data acts as marker for the arterial wall oxygen depletion with respect to geometrical parameters.

From Figures 7–9, in general, the results show that increasing l , which corresponds to increasing the length of the ILT, causes a more uniform oxygen distribution on the outer surface of the arterial wall, while it does not affect the oxygen measurements and concentration patterns within the ILT in a sensitive manner. Decreasing l , which decreases the length of the ILT, slightly affects uniformity of the diffusion and reduces the concentration on the regions that are not covered by the shorter ILT significantly. We also observe that in contrast to l , increasing δ which correspond to increasing the thickness of the ILT has a profound effect on the oxygen distribution of the wall as well as concentration patterns within the ILT, decreases oxygen flux to the wall and depletes oxygen within the ILT at the same time. More precisely, the minimum oxygen concentration values along the inner wall interface corresponds with ILT location. Therefore, the flux on the outer surface of the arterial wall has the largest change at the ILT region. We can clearly see that thicker ILT causes a decrease in oxygen supply to the outer surface of ILT, and therefore arterial wall as well. This trend can be noticed in two-level concentration contour plots, where low oxygen regions (blue area) are larger than the red high oxygen regions. Thus, the thicker ILTs influence greater reduction of oxygen diffusion from blood flow to the inner AAA wall. These observations are in agreement with the results of Figures 3–5. The attenuation of oxygen flux due to increasing l and δ is particularly apparent for the case (8,1) in *BD*= 4.2 cm which has the longest and thickest ILT between all the cases. The oxygen supply from the lumen to the inner layer of the wall in this case is blocked by the thick ILT and the ILT-wall interface is almost depleted of oxygen (Figure 7). Similarly, Figure 8 and Figure 9 suggest that the thicker and longer ILTs inhibit the oxygen transport to the arterial wall, as oxygen concentration near the lateral bulges of the ILT is significantly lower than the medial portions of the ILT. Finally, increasing *BD*, which corresponds to the severity of the aneurysm and increases the surface curvature in the ILT region, does not seem to affect oxygen measurements significantly according to Figures 7–9, as all the contours for oxygen diffusion in AAA wall and ILT follow relatively the same patterns for each case (l,δ) at different *BD* values.

**FIGURE 10:**
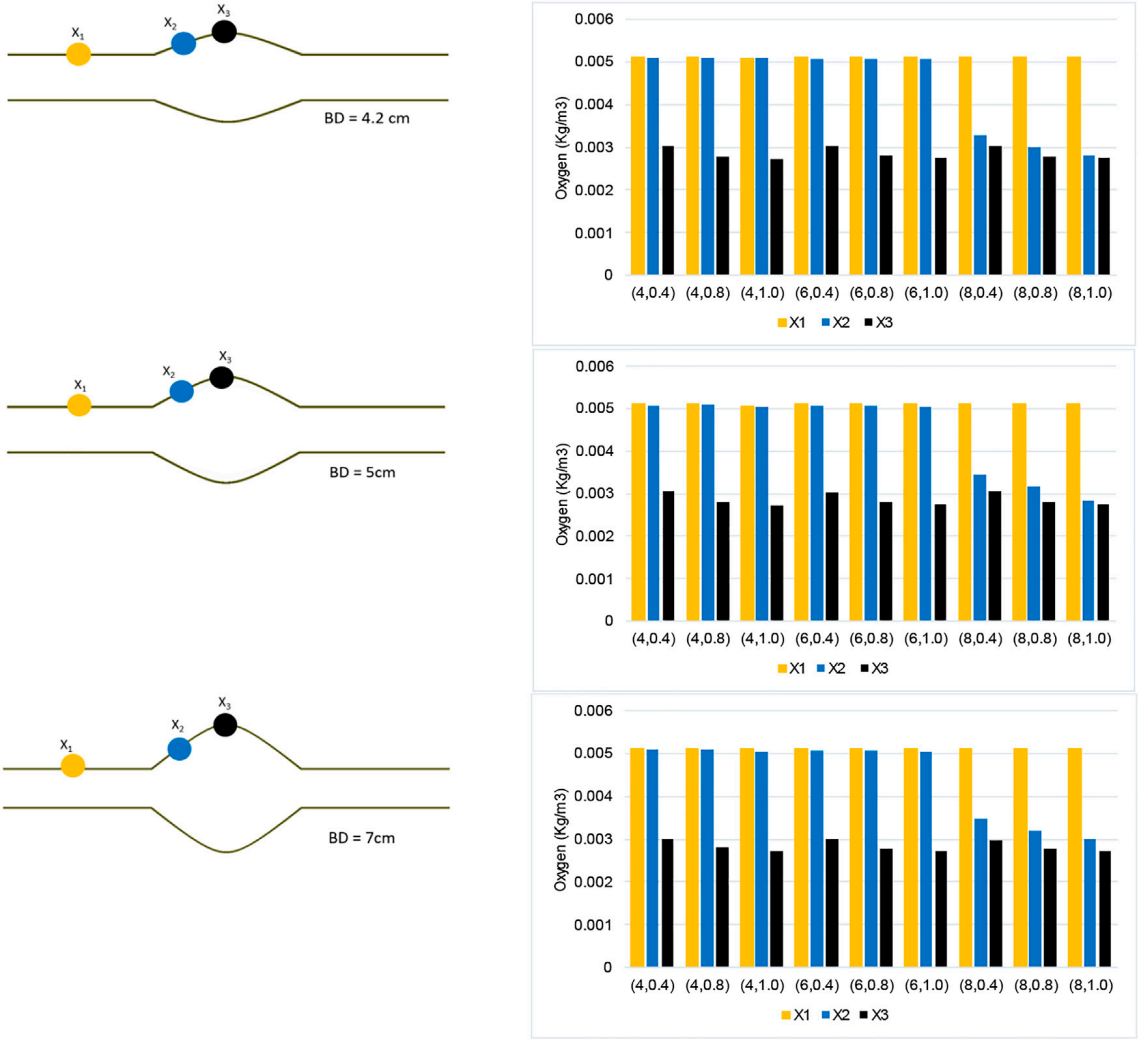
Oxygen concentration data collected along three points of the AAA model for each case (l,δ) , where l denotes ILT length and δ is ILT thickness. Plots corresponds to *BD*= 4.2 cm (top), *BD*= 5 cm (middle), and *BD*= 7 cm (bottom), respectively.

Figure 10 analyzes the spatial distribution of the oxygen concentration on the inner AAA wall surface by collecting and comparing the concentration values at each of the three displayed points. Oxygen concentration data for varying *BD* cases are provided. Point $X_{1}$ is located on the inner wall interface before the bulge, at the straight portion of the vessel that thrombus does not exist. This point $X_{1}$ has the highest oxygen concentration value that is nearly equal to the blood flow concentration value $C_{f}^{in}.\;$ In general, the values at $X_{1}$ are constant between all different case (l,δ), since no  cases in this study have ILTs that cover this point. Points $X_{2}$ is taken at 9 cm form the AAA inlet, where will be covered by long ILT but not by the short and medium ILT. When the ILT does not cover this section of the vessel, the concentrations for $X_{2}\;$ remains almost similar to $X_{1}$ , but is lower due to the bulging of the vessel. However, concentrations values significantly change when the ILT is long enough to cover that area of the vessel. Therefore, here is a significant drop in oxygen concentration at $X_{2}$ in the l = 8 cm ILT cases, while the cases with shorter ILTs of l = 4 cm and l = 6 cm do not cover $X_{2}$ location.

Moreover, point $X_{3}$ is located at the maximum diameter of the AAA, *BD*, which occurs at halfway along the length and has the highest thickness of ILT (see Figure 1 for a schematic illustration of the model). As the ILT grows in thickness, the oxygen attenuation causes concentration values to decrease at this location. Similar to previous observations (Figures 7–9), Figure 10 also asserts that the bulge diameter of AAA does not account for significant variation in arterial wall oxygen distribution, and therefore hypoxia. This is because the data follows the same rough pattern of oxygen concentration among different ILT morphologies for varying bulge diameter values.

**FIGURE 11:**
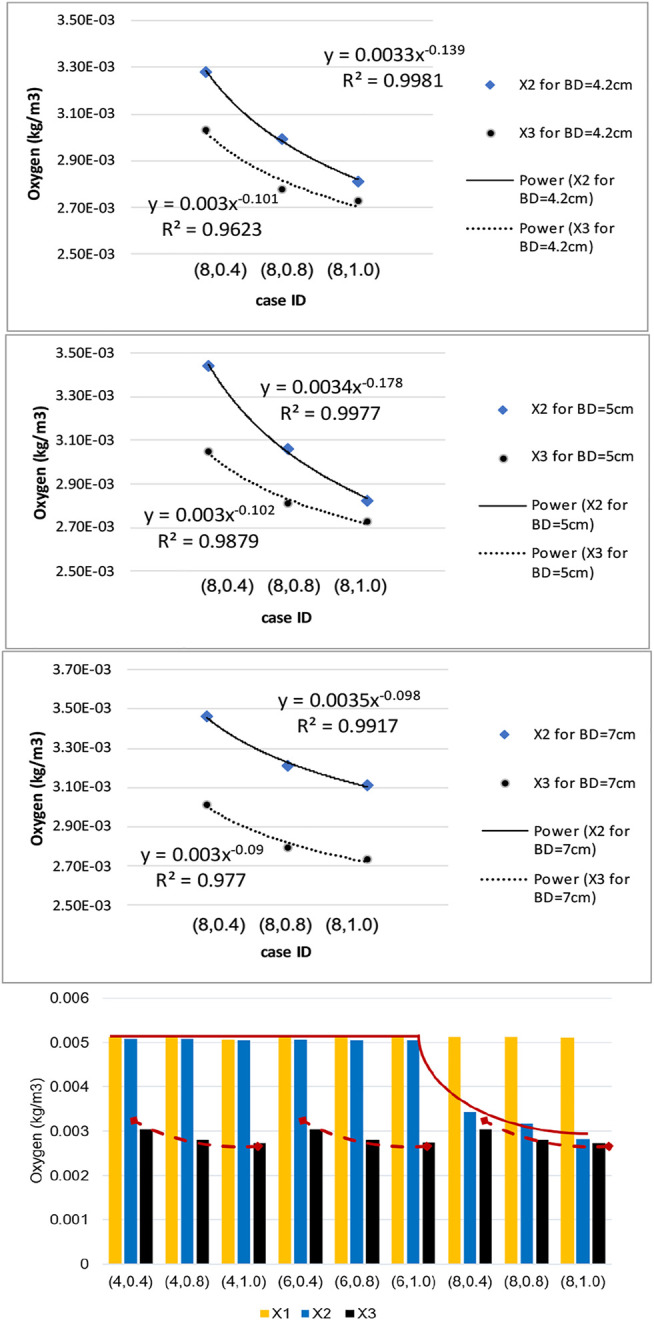
Sensitivity results for dependence of wall oxygen concentration to the model parameters δ and l , and the schematic of the measuring indicator plots used in the analysis.

Using the numerical results presented in bar plots of Figure 10, we have studied the variation of oxygen concentration at the three selected points on the inner wall interface, when each geometric parameter, namely ILT length and thickness for a certain AAA bulge diameter, is varied individually. The outcome of the analysis is reported in Figure 11. For a better comparison of the different charts, concentration values are reported for the cases (l,δ=0.4,0.8,1)  in which ILT is long enough to cover point $X_{2}$ . To better clarify the effects of ILT thickness, data points corresponding to the $X_{2}$ concentration are denoted by blue diamonds, and data points corresponding to $X_{3}$ are shown in black circles.

We observe that the dependence of the oxygen concentration at $X_{2}$ and $X_{3}$ is nonlinear and can be described using a power law model, namely $y=c.x^{p}$ , where (*p*) is the exponent that quantifies the sensitivity of the quantity of interest (*y*) with respect to the control parameter (*x*) and c is a scaling constant. Since the $X_{2}$ and $X_{3}$ data for different bulge diameters demonstrates a similar trend, we have fitted them using the same power law functional relationship. We observe that all the charts show a similar behavior and that the scaling constants are comparable, but the exponents of the power law are slightly different (Figure 11). In particular, exponent p is a little higher for point $X_{3}$ at different bulge diameters. Oxygen concentrations in $X_{3}$ are affected by ILT thickness, while the concentration values at $X_{2}$ are affected by ILT length and only vary if ILT is long enough to cover that point. The length of the ILT had a direct relationship with increase in local ILT thickness at the lesion, and therefore the computer model demonstrated an inverse relationship of wall oxygen measures at the point $X_{2}$ that is covered by the length of ILT with ILT thickness.

## Discussion

The objective of this study is to quantify the key geometric parameters which cause fluctuations in oxygen transport that might lead to oxygen deprivation in the arterial wall, and to investigate if there is a relation between wall hypoxia and ILT size in AAA. The numerical experiments are designed to quantify the influence of ILT length and thickness on AAA hypoxia. In addition, we simulated varying AAA bulge diameters to confirm our findings during different stages of AAA development. Analysis is performed to model fluctuations in oxygen transport, and the oxygen concentration profiles at different locations within an AAA model are presented and discussed.

Computer simulations predicted a gradient in oxygen concentration through the arterial wall, in particular in the region that is covered by the ILT, with maximum concentration value at the thrombus-free position, intermediate value at a medial region, and lowest value at the AAA wall (Figure 3). Therefore an ILT can decrease oxygen diffusion from the bloodstream to the underlying ILT and aneurysmal wall which is in agreement with observations in (Wang et al., 2002). The obtained results in Figures 4–6 have shown that concentration values along ILT abluminal surface and within the ILT tissue are highly influenced by the ILT thickness: the thinner the ILT, the greater the oxygen concentration measurement. Moreover, the results have highlighted that AAAs with longer ILT demonstrate a more uniform oxygen distribution pattern.

Comparison of contour plots for oxygen flux to tunica adventitia layer and oxygen concentration in the ILT of all case (l,δ) geometries at different *BD* values indicates that the degree of oxygen attenuation in the wall is closely related to the ILT thickness. In the wall, there is a clear gradient of oxygen, and the flux is negatively correlated with ILT thickness. More precisely, results demonstrated that luminal oxygen diffusion through the ILT to the AAA wall decreases significantly with increasing ILT thickness, resulting in low oxygen concentration at the inner wall surface (Figure 7, Figure 9 and Figure 8). This suggests that localized hypoxia occurs in AAA with thicker ILTs, and the thickness of the ILT layer has a profound effect on the oxygen concentration within AAA. The obtained behavior for oxygen concentration profiles were consistent with observations at Vorp et al. study (Vorp et al., 1998). Moreover, data presented in two-level oxygen contour plots (Figures 7–9) confirms that thicker and longer ILTs inhibit oxygen diffusion the most. This is due to the fact that thicker and longer ILTs take up more surface area and volume than other ILTs, therefore, less arterial wall surface is exposed to blood flow, and oxygen concentration to these covered areas is reduced. The thick and long cases have inflection points that are relatively more medial than their thin-and-short-ILT counterparts.

The bar plots of oxygen concentration values at different location of the wall inner surface for analyzed geometries (Figure 10) support this claim that ILT thickness attenuates oxygen flow most, as oxygen concentration gradually decreases as ILT thickness increases for every length of ILT. Figure 10 also demonstrates that the diameter of the AAA bulge has minimal effect on the oxygen concentration within the arterial wall. We observe that variations in oxygen diffusion follow a similar distribution among all bulge diameter plots (Figure 10). Since concentration values at $X_{3}$ are directly related to ILT thickness, it is clear that the ILT thickness impacts oxygen concentration at the arterial wall most, followed by noticeable sensitivity to the ILT length (Table 3; Figure 11). More precisely, increasing ILT length leads to increase in local ILT thickness at the same time at the affected region, and therefore decreases wall oxygen concentration.

**TABLE 3:** Exponent of the power law for different points of the sensitivity analysis.

Point	Bulge diameter (cm)	Value of the exponent ( p ) in $y=c.x^{-p}$
$X_{3}$	4.2	0.139
	5	0.178
	7	0.098
$X_{2}$	4.2	0.101
	5	0.102
	7	0.090

In summary, the arterial wall oxygen deprivation is nearly independent of aneurysm bulge diameter and depends only on the geometrical features of the ILT layer. The ILT thickness and wall oxygen concentration are anti-correlated, while the variation in ILT length affects the local thickness of the ILT and evens the oxygen distribution throughout the arterial wall. As such, if we increase the axial length of the ILT, the result will be an increase the area of lowered oxygen concentration within aneurysmal wall. As hypothesized, our data suggests that ILT geometry variations can have the largest effect on wall oxygen deprivation. The thicker and longer ILTs reduce oxygen diffusion to the arterial wall regardless of bulge diameter and cause the most oxygen deprivation in AAA cases. Therefore, consideration of the ILT size and anatomy may be important in considering the severity of a particular AAA as recommended in (Riveros, 2013) and (Koole et al., 2013).

We would also like to point out that the high Sherwood number is a potential reason for low variation in oxygen diffusion among cases with different bulge diameters. The flow in the lumen is advection dominated and the time scale characteristic of the blood flow is too short to allow fluid movement to take place between material elements by diffusive mass transport. This indicates that bulge diameter affects oxygen flow insignificantly. Therefore, the mass transport is AAA geometry independent and more ILT geometry dependent. Instabilities in the flow can occur for certain ranges of Reynolds number and AAA severity.

The numerical methodology was validated using numerical research data of the literature, as described in Zakerzadeh et al. (Zakerzadeh et al., 2021). The blood flow with the velocity of  v≈ 0.23 m/s matches physiological Reynolds numbers and represent the flow in the abdominal aorta under the resting condition (Fraser et al., 2008). We compared the results of wall oxygen concentration obtained from *in-situ* mass transport with experimental data for hypoxia in (Vorp et al., 2001) where normalized partial pressure of oxygen in the ILT-wall interface as well as a random point inside the AAA wall for large group of patients with thin and thick ILT has been reported. We have observed a qualitative consistency with these experimental results. However, it would be extremely difficult to find suitable clinical or *in vitro* data for quantitative validation, since precise details on variation in wall thickness, ILT thickness, and presence of vasa vasorum can significantly affect the results. As for numerical verification, mesh convergence analysis is performed (see (Zakerzadeh et al., 2021) for details of mesh independence study). The obtained oxygen concentration profiles tested against these Vorp et al.‘s data (Vorp et al., 1998), and the qualitative nature of the oxygen concentration is in agreement.

Our approach, however, has some limitations. Principle among these is that a simplified geometry of idealized AAAs is used in this investigation. The idealized lesion geometries facilitate systematic variations of the three morphological features explored in this paper. In this way, we can more easily investigate the effect of these geometrical features on wall hypoxia, and thereby begin to build intuition otherwise not possible. We previously performed the analysis of combined flow and oxygen transport in a patient-specific AAA geometry (Zakerzadeh et al., 2020). However, patient-specific models prohibit creating a large number of parametric studies for the purpose of our study. In addition, to compare across AAAs, we do not have access to patient-specific blood flow rates. Nevertheless, the knowledge gained from the present work sets the ground for future research that manages patient-specific models. Moreover, the steady-state solution is obtained in the current study. The steady-state flow assumption gives a reasonable first approximation to time-averaged unsteady results, so long as Schmidt number is large (Ma et al., 1994). Given the blood oxygen diffusivity of 1.6 × 10^−9^ m^2^/s, the calculated Schmidt number is about 2,100, and therefore considering only steady conditions is an appropriate strategy for this study while significantly reducing the computational costs of fully three-dimensional mass transfer calculations. In fact, as the transient behavior has shown to have relatively minimal impact on lumen oxygen flux (Kolandavel et al., 2006; Liu et al., 2011), it will not significantly affect the flow-driven mass transport and the conclusions of this work. It has been established that ILT is heterogeneous and oxygen diffusion coefficient fluctuates in multiple ILT layers (Yunoki et al., 2012). Moreover, the arterial wall oxygen consumption rate varies spatially as well (Buerk and Goldstick, 1982). However, to our knowledge, these parameters have never been measured for different layers of an ILT. Since such an experimental study is outside the scope of the present work, we have assumed a value of these parameters from a compilation of previously published values for thrombus. However, these observations recommend that different ILT structural composition needs to be handled more cautiously in the future AAA simulations (see (Tong and Holzapfel, 2015) for a comprehensive research summary on ILT structure and mechanical characterization). Future improvements to our AAA models could also include integrating artificial intelligence (AI) and endothelial response into our workspace. By modeling the effect of a growing ILT on oxygen diffusion and wall stress using AI, we can better predict aneurysm rupture. Additionally, modeling the layers of the arterial wall and intraluminal thrombus tissues can improve our understanding of AAA formation and rupture at the cellular level. Coupling endothelial response data with a fluid-structure interaction (FSI) framework could also allow to track immune response to AAA formation and create drug delivery pathways to cure a clinical aneurysm. In attaining a higher-fidelity model, we also plan to include the pulsatile movement of the artery in future work. This would ask for an FSI framework. While such coupling may incur high computational cost, the potential of integrating AI and immune response into our FSI framework is promising. The proposed methodology is a step forward in the personalized medicine, quantifying the aneurysm rupture risk reduction, and helping the clinicians in the preoperative planning and making informed decisions towards effective treatment.

## Data Availability

The original contributions presented in the study are included in the article/Supplementary Material, further inquiries can be directed to the corresponding author.
